# Identification of a Chinese patient with *MTSS2*-causing intellectual disability by WES reanalysis: a case report

**DOI:** 10.3389/fpsyt.2025.1707837

**Published:** 2026-01-20

**Authors:** Duan Li, Wen Zhang, Huifen Mei, Xiaodan Chen, Zhikun Lu, Pengfei Wu, Li Liu, Yunting Lin

**Affiliations:** Department of Genetics and Endocrinology, Guangzhou Women and Children’s Medical Center, Guangzhou Medical University, Guangdong Provincial Clinical Research Center for Child Health, Guangzhou, China

**Keywords:** intellectual disability, *MTSS2* gene, *MTSS1L* gene, whole exome sequencing, WES reanalysis

## Abstract

Intellectual developmental disorder with ocular anomalies and distinctive facial features (IDDOF) is an extremely rare disease caused by a heterozygous pathogenic variant in the *MTSS2* gene with an autosomal dominant inheritance pattern. To date, only 10 patients with IDDOF and one pathogenic variant in the *MTSS2* gene have been reported. Here, we present a new Chinese patient with IDDOF, who is the 11th patient worldwide and the second case in China. The proband presented with relative microcephaly, distinctive facial features of bitemporal narrowing and ptosis, ophthalmological and auditory findings, hypotonia, psychomotor developmental delay, intellectual disability, and emotional and behavioral problems. Whole exome sequencing (WES) initially did not find a phenotype-contributing variant in 2021, whereas reanalysis of WES data in 2024 revealed that the *de novo* c.2011C>T(p.Arg671Trp) heterozygous variant of the *MTSS2* gene in the patient turned out to be pathogenic, which had been reported to cause IDDOF in 2022. Our study reports an additional patient and new phenotypes for IDDOF and suggests the c.2011C>T(p.Arg671Trp) variant in the *MTSS2* gene as a hotspot. Our work shares our diagnostic experience and indicates the potential of WES reanalysis in negative cases to improve diagnostic yield, particularly in rediscovering previously unknown gene–disease associations.

## 1 Introduction

Intellectual disability (ID) is one of the most prevalent neurodevelopmental disorders affecting 1%-3% of the general population (1). To date, more than 2,000 genes have been implicated in ID with high genetic heterogeneity and phenotypic variability. Therein, *de novo* variants with autosomal dominant trait are prominent contributors to ID in non-consanguineous families (2).

The *MTSS2* gene (OMIM * 615951), formerly known as *MTSS1L* gene, encodes MTSS I-BAR domain-containing protein 2, which is mainly expressed in the central nervous system (3). In 2022, a recurrent *de novo* c.2011C>T(p.Arg671Trp) variant in the *MTSS2* gene (NM_138383.3) was revealed to cause a novel ID syndrome in five unrelated individuals through a dominant-negative mechanism (4). This associated disorder is now named “Intellectual developmental disorder with ocular anomalies and distinctive facial features (IDDOF)” (OMIM # 620086) in the Online Mendelian Inheritance in Man (OMIM) database. Subsequently, an additional case of IDDOF was reported in 2023 (5). Recently, four new cases have been added to the literature (6). However, the mutational spectrum of this disorder has not expanded, since all 10 reported patients share the same *MTSS2* c.2011C>T(p.Arg671Trp) variant.

Here, we present a new Chinese boy with IDDOF, who is the 11th patient worldwide and the second case in China, and describe his diagnostic process.

## 2 Case presentation

**Figure 1:**
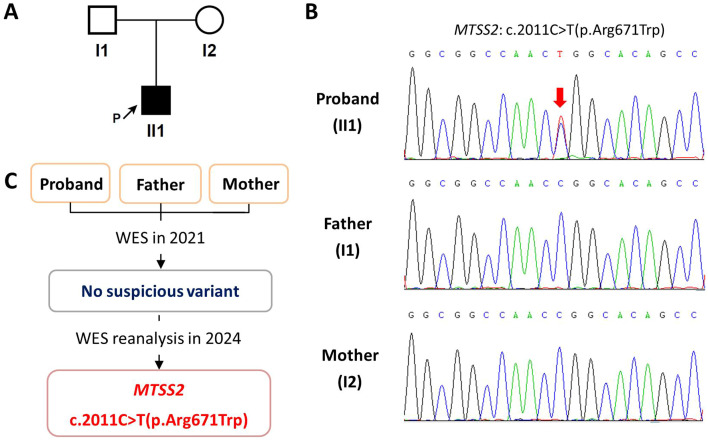
The family pedigree and molecular findings of the enrolled family. **(A)** The family pedigree. **(B)** Sanger sequencing chromatograms of the enrolled family. The red arrow indicates the variant. **(C)** The molecular diagnostic process of the proband.

The proband (II1) is the first child of the non-consanguineous healthy parents (I1 and I2) (**Figure 1A**). He was delivered naturally at term with a normal length of 51 cm (0.3 SD) and weight of 3.6 kg (0.7 SD) (7). There was no history of prenatal issues or birth injury. After birth, the proband was noticed to have ptosis and hypotonia and gradually developed psychomotor delay. He achieved head control at 4 months of age, could turn over at 8 months of age, started walking independently at 1 year and 10 months of age, and enabled to speak until 2 years and 7 months of age. His brain magnetic resonance imaging at 7 months of age and electroencephalogram at 2 years and 2 months of age had no obvious abnormality. His skeletal X-ray at 1 year and 6 months of age indicated spina bifida occulta of the fifth lumbar vertebra, and auditory evoked potential at 2 years and 3 months of age showed a slight increase of bilateral hearing thresholds.

The proband first presented to our clinic at 2 years and 7 months of age in 2021 because of ID, speech delay, and motor incoordination. He presented with distinctive facial features of bitemporal narrowing and ptosis. He had a normal height of 93.2 cm (−0.2 SD) and a weight of 14.5 kg (0.4 SD) but relative microcephaly with an occipitofrontal circumference of 47.5 cm (−1.4 SD) and chest circumference of 51 cm (0.1 SD) (7, 8). Physical examination found a small cafe-au-lait spot on his left thigh and an enlargement of his left scrotum. Ophthalmological examination revealed astigmatism and myopia. Furthermore, the proband suffered from emotional and behavioral problems, such as bad temper, irritability, and automatic speaking. No abnormality was found in his biochemical and metabolic profiles, karyotype analysis, and chromosomal microarray analysis.

To identify the phenotype-producing variant, whole exome sequencing (WES) of the proband–parent trio was performed. The analysis identified several variants: a *de novo* variant in the *MTSS1L* gene; two hemizygous variants in the *BCORL1* and *IGSF1* genes, both inherited from the mother; and a compound heterozygous variant in the *AHNAK2* gene, inherited from each parent (**Table 1**). Among them, only *BCORL1* and *IGSF1* were known disease-causing genes, but the identified variants in these two genes were predicted to be tolerated by in silico analyses (**Table 2**). Therefore, none of the identified variants could explain the proband’s phenotypes.

**Table 1:** Identified variants of the proband based on family model in 2021.

Family model	Gene	Chr	Transcript	Variant	Allele	Inheritance	OMIM-disease
Dominant	*MTSS1L*	16	NM_138383	c.2011C>T(p.Arg671Trp)	Het	*De novo*	–
Recessive	*BCORL1*	X	NM_021946	c.3422G>A(p.Arg1141Gln)	Hem	Maternal	Shukla-Vernon syndrome (XLR)
Recessive	*IGSF1*	X	NM_001170961	c.1793A>T(p.Glu598Val)	Hem	Maternal	Hypothyroidism, central, and testicular enlargement (XLR)
Compound heterozygous	*AHNAK2*	14	NM_138420	c.5531C>T(p.Ala1844Val) c.9850A>C(p.Lys3284Gln)	Het Het	Paternal Maternal	–

Chr, chromosome; Het, heterozygous; Hem, hemizygous; XLR, X-linked recessive; -, none.

**Table 2:** In silico analyses of the *BCORL1* and *IGSF1* variants.

Variant	PROVEAN	SIFT	PolyPhen-2	Mutation taster	FATHMM
*BCORL1*: c.3422G>A(p.Arg1141Gln)	Neutral	Tolerated	Possibly damaging/benign	Benign	Tolerated
*IGSF1*: c.1793A>T(p.Glu598Val)	Neutral	Tolerated	Possibly damaging/possibly damaging	Benign	Tolerated

The proband was then lost to follow-up until 5 years and 3 months of age when he revisited our clinic for further examination. He remained with a normal height and weight, and relative microcephaly with an occipitofrontal circumference of 49 cm (−1.7 SD) and chest circumference of 55 cm (−0.4 SD) (8). There was no significant improvement for his neurological phenotypes of ID, speech delay, and motor incoordination.

To further investigate if there is an underlying molecular basis, WES data were reanalyzed in 2024. Surprisingly, the *de novo* c.2011C>T(p.Arg671Trp) heterozygous variant of the *MTSS1L* gene, now known as *MTSS2* gene, in the proband turned out to be a known pathogenic variant of IDDOF. Sanger sequencing subsequently confirmed the *de novo* status of this variant with presence in the proband and absence in his unaffected parents (**Figure 1B**).

## 3 Discussion

IDDOF is an extremely rare disease caused by a heterozygous deleterious variant in the *MTSS2* gene with an autosomal dominant inheritance pattern. To date, only 10 patients with IDDOF and one pathogenic variant in the *MTSS2* gene have been reported (4–6). Our case shared similar facial appearances and neurologic features, and the same c.2011C>T(p.Arg671Trp) heterozygous variant in the *MTSS2* gene with these previous patients (**Table 3**), which further supports the *MTSS2* gene as the cause of IDDOF and suggests the c.2011C>T(p.Arg671Trp) variant in the *MTSS2* gene as a hotspot. Thus, our patient could be diagnosed as IDDOF clinically and genetically.

**Table 3:** Clinical and genetic features of 11 patients with IDDOF.

Patient	P1	P2	P3	P4	P5	P6	P7	P8	P9	P10	P11	Mean ± SD or percentage
Reference	Huang Y et al. (3)					Corona-Rivera JR et al. (6)	Dominicis A et al. (7)				This study	/
Gender	Male	Female	Male	Male	Male	Male	Male	Female	Male	Male	Male	Male:female = 9:2
Ethnicity	European	UK	Dutch	European	Chinese	Mexican	European	European	European	European	Chinese	/
Age (years)	8	42	15	1.17	1.75	7	10	21	7.67	12	5.25	8.8 ± 5.7
Growth retardation	−	−	Unknown	−	−	+	−	+	−	−	−	20.0%(2/10)
Facial dysmorphism	+	+	+	+	Unknown	+	+	+	+	+	+	100.0%(10/10)
Microcephaly or relative microcephaly	+	+	+	+	+	+	+	+	+	+	+	100.0%(11/11)
Ophthalmological anomalies	+	+	+	+	+	+	+	−	+	−	+	81.8%(9/11)
Hearing impairment	+	+	Unknown	−	−	Unknown	−	+	−	−	+	44.4%(4/9)
ID	+	+	+	+	+	+	+	+	+	+	+	100.0%(11/11)
Autism spectrum disorder	+	−	+	Not applicable	Not applicable	+	−	−	−	−	−	33.3%(3/9)
Seizures	−	+	−	−	−	+	+	+	−	−	−	36.7%(4/11)
Gene variant	*MTSS2*: c.2011C>T(p.Arg671Trp) heterozygous variant											/
Inheritance	*De novo*	*De novo*	*De novo*	*De novo*	*De novo*	Unknown (absence in his co-twin and mother)	*De novo*	*De novo*	*De novo*	Unknown	*De novo*	/

However, the confirmation of IDDOF did not change the supportive rehabilitation interventions for our patient, as there is no effective treatment for this disease. For his parents, prenatal diagnosis was suggested for future pregnancies because gonadal mosaicism could not be ruled out, even though the pathogenic *MTSS2* variant was not detected in their blood samples.

In addition to the classical symptoms of IDDOF, our patient exhibited previously unreported phenotypes, including spina bifida occulta of fifth lumbar vertebra, an enlarged scrotum, and astigmatism. Among the 10 previously reported IDDOF patients, some showed ophthalmological manifestations such as foveal hypoplasia, progressive optic nerve atrophy, iris cysts, and myopia, while the patient from Mexico had bilateral inguinal hernias and bilateral cryptorchidism (5–7). Moreover, recent studies have implicated the MTSS2 protein in cell migration and spinogenesis (9) and have suggested that variants in the *MTSS2* gene may contribute to the etiopathogenesis of spina bifida (10). Therefore, these findings collectively suggest that spina bifida and ophthalmological and genitourinary abnormalities are specific features of IDDOF.

In this study, our initial WES analysis in 2021 did not find the phenotype-causing variant in the proband, whereas the reanalysis in 2024 successfully identified it (**Figure 1C**). That is because the *MTSS2* gene was first described to cause IDDOF in 2022 (4). Our initial WES analysis based on OMIM in 2021 could not recognize this novel causative gene uncovered later. This study is very similar to our previous report on Marbach–Rustad progeroid syndrome resulting from the *de novo* c.1436C>T(p.Ser479Phe) heterozygous variant in the *LEMD2* gene (11). Our experience indicates that WES reanalysis of negative cases could improve diagnostic yield, particularly when there are previously unknown gene–disease associations.

Actually, our initial identification of the *de novo* c.2011C>T(p.Arg671Trp) variant in the *MTSS2* gene in 2021 was earlier than the first gene-disease report in 2022. However, the lack of resources for further functional studies, coupled with limited access to genomic matchmaking platforms and inter-institutional collaboration, prevented us from identifying this new disease-causing gene.

In addition, although only the c.2011C>T(p.Arg671Trp) variant in the *MTSS2* gene has been confirmed as the cause of IDDOF, 18 different variants in the *MTSS2* gene have been documented in the Human Gene Mutation Database (Professional 2025.2). Among them, 13 variants are annotated with presence in patients having neurodevelopment disorders.

In fact, the *MTSS2* gene was first described as a novel candidate gene for human neurogenetic disorders in 2015 as the c.1790C>T(p.Thr597Met) missense variant was found in two siblings presenting ID and segregated in their family with an autosomal recessive inheritance pattern (12). However, the clinical significance of this c.1790C>T(p.Thr597Met) variant needs to be further evaluated due to a high frequency of 1.6% recorded in the Kinh Vietnamese genetic variation database (13). Thereafter, several variants in the *MTSS2* gene were found in patients affected by developmental disorder or autism, but their clinical significances were undefined (14–17). More studies are needed to determine whether there are novel inheritance pattern and more pathogenic variants for IDDOF.

## 4 Conclusions

Our study adds a new patient with IDDOF, who is the 11th case worldwide and the second case in China, and provides additional phenotypes to expand the phenotypic spectrum of this disease. Our finding supports the *MTSS2* gene as the cause of IDDOF and indicates the c.2011C>T(p.Arg671Trp) variant in the *MTSS2* gene as a hotspot. Our experience also underlines the potential of WES reanalysis to improve diagnostic efficiency, particularly in rediscovering previously unknown gene–disease associations.

## Data Availability

The data that support the findings of this study are available from the corresponding author upon reasonable request.
